# Factors influencing smartphone overdependence among adolescents

**DOI:** 10.1038/s41598-024-58152-1

**Published:** 2024-04-02

**Authors:** Dabok Noh, Mi-So Shim

**Affiliations:** 1 College of Nursing, Eulji University, Seongnam, Republic of Korea; 2 College of Nursing, Keimyung University, Daegu, Republic of Korea

**Keywords:** Adolescents, Anxiety, Depression, Loneliness, Smartphone overdependence, Human behaviour, Risk factors

## Abstract

Adolescents are particularly vulnerable to smartphone overdependence. Therefore, we identified the factors influencing smartphone overdependence and risk subgroups among adolescents. The current study is a secondary analysis of nationally representative data from the 2020 Korea Youth Risk Behavior Survey. The survey targeted middle- and high-school students in South Korea aged 12–18 using stratified, clustered, multistage probability sampling, and 53,457 students from 793 schools participated in this study. Complex sample data were analyzed considering the strata, clusters, and weights. Multiple logistic regression analysis revealed age, gender, household economic status, anxiety, loneliness, depressive symptoms, and experience of violent treatment as significant predictors of smartphone overdependence. Adolescents with severe anxiety were at a 3.326 times higher risk of smartphone overdependence than adolescents with minimal anxiety. Decision tree analysis showed that anxiety, gender, loneliness, and depressive symptoms were important in differentiating risk subgroups, with anxiety being the most significant factor. Group 13, comprising girls with severe anxiety, had the highest risk at 52.9%. Thus, early detection and prevention of issues such as anxiety, loneliness, and depressive symptoms, as well as treatment for violence, can prevent smartphone overdependence among adolescents. Additionally, more thorough interventions for anxiety are warranted to prevent smartphone overdependence.

## Introduction

For adolescents, smartphones have transcended their role as media technology (used when necessary) and become a cultural tool employed to reorganize social relationships and interact with others^1^. With the increasing use of smartphones, overdependence has become a global concern^2^. According to a South Korean national survey, 23.3% of individuals aged 3–69 were in the smartphone overdependence group in 2020—a rapid increase from 20.0% in 2019^3^. Adolescents are particularly vulnerable to smartphone overdependence^4^; South Korea is no exception, with smartphone overdependence in the country being more prevalent among adolescents than among children and adults^3^. Similarly, in Switzerland, the incidence of smartphone addiction was found to be significantly higher in adolescents aged 15–16 than in young adults aged ≥ 19 years^5^.

Smartphone overdependence refers to a state involving three characteristics: increased salience of smartphone use, uncontrolled smartphone use, and serious consequences^6^. In this definition, “salience” alludes to the state in which smartphone use becomes the most important activity, as smartphone stimuli (e.g., content or usage environment) are perceived more prominently than other stimuli. Furthermore, “uncontrolled” refers to a state in which a user’s self-regulatory ability is insufficient for meeting their subjective goals, and “serious consequences” pertains to negative physical, interpersonal, and social outcomes^6^.

Problematic smartphone use has shifted from addiction to overdependence. As the addiction framework mainly focuses on symptoms such as withdrawal and tolerance, its application to problematic smartphone use, which has a sociocultural component, is difficult^6^. Therefore, problematic smartphone use should be separated from the addiction framework^7^. Considering this evidence, we use the term “smartphone overdependence” in the current study.

According to the cognitive–behavioral model of pathological Internet use^8^, factors associated with problematic Internet use include anxiety, depression, social isolation, and lack of social support. This model can also be applied to the current phenomenon of smartphone overdependence. Anxiety and depression have been found to be associated with smartphone overdependence among Chinese adolescents^9^ and university students in Lebanon^10^. During the coronavirus disease 2019 (COVID-19) pandemic, loneliness due to social isolation emerged as an important risk factor for smartphone overdependence^11^. Loneliness was also found to be linked to smartphone overdependence among adolescents in Turkey^12^ and Indonesia^13^. Additionally, adolescents who experience violence perpetrated by parents and/or peers are exposed to a lack of social support, which affects smartphone overdependence^14,15^. Thus, anxiety, loneliness, depression, and experience of violence have been associated with smartphone overdependence.

Although studies have been conducted on smartphone overdependence among adolescents, research identifying subgroups at high risk is lacking. Therefore, in this study, decision tree (DT) analysis was performed along with analyzing the influencing factors of smartphone overdependence using logistic regression. DT analysis can confirm the interaction of various variables and identify risk subgroups, providing results that can be used for classification in the healthcare field^16^. In previous studies, DT analysis has been performed to identify risk groups for Internet overdependence among adults^17^ and Internet game addiction among adolescents^18^. It was also applied to the analysis of adolescents’ smartphone addiction in one study, but the study was limited in that it did not include various psychological factors^19^. Thus, we aimed to (1) identify factors influencing smartphone overdependence among adolescents, including anxiety, loneliness, depressive symptoms, and experience of violence and (2) derive subgroups at risk of smartphone overdependence through DT analysis.

## Material and methods

### Design

We performed a secondary data analysis using a national cross-sectional Web-based survey dataset obtained from the 16th Korea Youth Risk Behavior Survey (KYRBS); the data were collected from August to November 2020 and established as reliable by Statistics Korea^20^. KYRBS data are made public annually via the Korea Disease Control and Prevention Agency website^21^.

### Setting and participants

The survey targeted middle- and high-school students in South Korea aged 12–18 using stratified, clustered, multistage probability sampling^21^. The total population was divided into 117 strata based on administrative divisions and school type, while 400 middle schools and 400 high schools were selected using stratified random sampling. From the selected schools, a classroom in each grade was randomly sampled, and all students in the selected classes were recruited.

Standardized operational procedures were designed to protect the voluntary participation and anonymity of the adolescents^21^. Teachers trained by the Korea Disease Control and Prevention Agency, excluding homeroom teachers, explained the purpose of the study and the participation procedure. The adolescents anonymously completed a self-administered Web-based questionnaire in a school computer laboratory during class. Of the initial sample of 57,925 students from 800 schools, 54,948 students from 793 schools participated in the survey, yielding a response rate of 94.9%. As the dependent variable was smartphone overdependence, we checked the responses to the question regarding whether smartphones were used on weekdays or weekends, to exclude participants who did not use smartphones. Resultantly, a final total of 53,457 participants were included in our analysis, after excluding 1491 who stated that they did not use smartphones on both weekdays and weekends.

### Measures

The sociodemographic characteristics included age, gender, and household economic status (high, medium, and low). Anxiety, loneliness, depressive symptoms, and the experience of treatment were included as independent variables^21^.

Anxiety was measured using the seven-item Generalized Anxiety Disorder (GAD-7) scale^22^. Respondents were asked to rate how often they had experienced anxiety-related feelings (e.g., feeling nervous, anxious, and on the edge) over the last two weeks on a 4-point Likert scale. The total scores ranged from 0 to 21, with higher scores indicating higher levels of anxiety. This tool provides the following cutoff scores: 0–4 (minimal anxiety), 5–9 (mild anxiety), 10–14 (moderate anxiety), and 15–21 (severe anxiety). The Cronbach’s alpha coefficients of the GAD-7 were 0.93 and 0.90 in Ahn et al.’s study^23^ and this study, respectively.

Loneliness was assessed by asking adolescents to respond to the question “How often have you felt lonely during the last 12 months?” on a 5-point Likert scale^21^. Higher scores indicated higher levels of loneliness; subsequently, the scores were converted into a dichotomous variable (“0” = none or sometimes and “1” = often or always).

Depressive symptoms were assessed using the question “Were you feeling so sad or hopeless that you stopped doing certain usual activities for at least two weeks or more during the last 12 months?”^21^ The response options were yes and no.

The experience of treatment necessary as a result of violence was measured with the question “In the past 12 months, have you been treated in a hospital because of violence (physical assault, intimidation, bullying, etc.) perpetrated by a friend, colleague, or adult?”^21^ The frequency was measured as “0 times” when there was no treatment owing to violence and “1–6 or more times” when there was experience of treatment owing to violence. Responses were converted into a dichotomous variable (“0” = no experience of treatment owing to violence and “1” = having experience of treatment owing to violence).

Smartphone overdependence was assessed using the Smartphone Overdependence Scale for adolescents^6^. This tool comprises 10 items rated on a 4-point Likert scale, with total scores ranging from 10 to 40. An example item is “It is difficult to control the usage time of smartphones.” The total scores were categorized into two groups (general and risk) based on a score of 23, which was the threshold for dependence^6^. The Cronbach’s alpha coefficients were 0.84 and 0.92 in the previous national report^6^ and this study, respectively.

### Statistical analysis

Complex sample data were analyzed considering strata, clusters, and weights using SPSS Statistics for Windows version 27 (IBM Corp., Armonk, NY, USA). Frequency analysis and descriptive statistics were used to obtain the univariate summary statistics of the variables. The results were summarized as counts and weighted percentages or weighted means and standard errors. Bivariate analyses comparing the independent variables (sociodemographic characteristics, anxiety, loneliness, depressive symptoms, and experience of treatment owing to violence) and the dependent variable (presence of smartphone overdependence) were conducted by performing independent t-tests and chi-square tests. Subsequently, complex sample multiple logistic regression analysis was employed to conduct a multivariate analysis of the predictors of smartphone overdependence. Finally, DT analysis was performed to identify risk subgroups for smartphone overdependence based on classification and regression trees. Classification and regression trees can be used for both categorical and continuous dependent variables and employ a Gini index or Twoing index to select input variables^16^. In this study, the minimum number of cases to be split was analyzed by specifying 1000 as the parent node and 500 as the child node.

### Ethical considerations

The KYRBS is conducted annually to investigate the health status and behavior of adolescents in South Korea^24^. As the KYRBS is conducted without institutional review board approval based on the Enforcement Regulations of the Bioethics and Safety Act of Korea, written consent was not required from the participants and/or their legal guardians^24^. Before this study, however, all participants were given an explanation about its purpose, procedure, and contents and engaged voluntarily, filling out an online informed consent form. Personal identification information was not collected during the survey. This study was approved by the Eulji University Institutional Review Board (registration number EUIRB2023-049), and all methods were in accordance with the guidelines and regulations of the Declaration of Helsinki.

## Results

### Summary statistics of variables

The mean age of the participants was 15.17 years. This study included a slightly higher proportion of boys (51.2%) than girls (48.8%). Almost 50% of the participants reported medium household economic status. The mean anxiety score was 3.95, with 22.3%, 7.7%, and 3.5% of the participants experiencing mild, moderate, and severe anxiety, respectively. The proportion of participants who felt lonely often or always was 14.1%, and 25.1% had depressive symptoms. The proportion of participants who experienced treatment owing to violence was 1.1%. The average smartphone overdependence score was 18.72, with 74.2% and 25.8% of participants in the general use and risk groups, respectively (Table 1).

**Table 1:** Sociodemographic and psychological characteristics of the participants (N = 53,457).

Variable	Categories	*n* (weighted %) or mean ± SE^a^
Age	–	15.17 ± 0.025
Gender	Boys	27,225 (51.2)
	Girls	26,232 (48.8)
Household economic status	High	20,565 (39.5)
	Medium	25,860 (47.9)
	Low	7032 (12.6)
Anxiety	Minimal	35,662 (66.5)
	Mild	11,859 (22.3)
	Moderate	4042 (7.7)
	Severe	1894 (3.5)
Loneliness	None or sometimes	45,918 (85.9)
	Often or always	7539 (14.1)
Depression	No	40,049 (74.9)
	Yes	13,408 (25.1)
Experience of treatment owing to violence	No	52,863 (98.9)
	Yes	594 (1.1)
Smartphone overdependence	General use group	39,864 (74.2)
	Risk group	13,593 (25.8)

^a^*SE* = standard error.

### Associations of sociodemographic and psychological characteristics with smartphone overdependence

Table 2 displays the independent t-test and chi-square test results for the independent variables and the dependent variable (smartphone overdependence). All independent variables were significantly associated with smartphone overdependence. The mean age of the smartphone overdependence risk group was higher than that of the general use group (t = 10.948, *p* < 0.001). The risk of smartphone overdependence was 30.2% in girls, significantly higher (*χ*^2^ = 519.717, *p* < 0.001) than in boys (21.6%). Participants with a low household economic status were more likely to be in the smartphone overdependence risk group (31.1%) than those with a high (23.6%) or medium household economic status (26.3%; *χ*^2^ = 158.219, *p* < 0.001). While 51.1% of participants with severe anxiety were in the smartphone overdependence risk group, participants with minimal anxiety exhibited the lowest risk of smartphone overdependence (18.5%; *χ*^2^ = 3195.350, *p* < 0.001). The risk of smartphone overdependence among participants who often or always felt lonely was 41.2%, significantly higher (*χ*^2^ = 1073.611, *p* < 0.001) than that among participants who did not feel lonely or felt lonely sometimes (23.3%). Participants with depressive symptoms were more likely to be at risk of developing smartphone overdependence (36.6%) than those without such symptoms (22.2%; *χ*^2^ = 1082.001, *p* < 0.001). Participants with experience of treatment owing to violence were more likely to be in the smartphone overdependence risk group (37.1%) than those without it (25.7%; *χ*^2^ = 39.822, *p* < 0.001).

**Table 2:** Prevalence of smartphone overdependence by sociodemographic and psychological characteristics (N = 53,457).

Variable	Categories	Smartphone overdependence, *n* (weighted %) or mean ± SE^a^		t/*χ*^2^ (*p*)
		General use group	Risk group	
Age	–	15.10 ± 0.026	15.36 ± 0.030	10.948 (< .001)
Gender	Boys	21,518 (78.4)	5707 (21.6)	519.717
	Girls	18,346 (69.8)	7886 (30.2)	(< .001)
Household economic status	High	15,793 (76.4)	4772 (23.6)	158.219 (< .001)
	Medium	19,173 (73.7)	6687 (26.3)	
	Low	4898 (68.9)	2134 (31.1)	
Anxiety	Minimal	29,176 (81.5)	6486 (18.5)	3195.350 (< .001)
	Mild	7518 (63.0)	4341 (37.0)	
	Moderate	2242 (55.0)	1800 (45.0)	
	Severe	928 (48.9)	966 (51.1)	
Loneliness	None or sometimes	35,385 (76.7)	10,533 (23.3)	1073.611 (< .001)
	Often or always	4479 (58.8)	3060 (41.2)	
Depression	No	31,308 (77.8)	8741 (22.2)	1082.001 (< .001)
	Yes	8556 (63.4)	4852 (36.6)	
Experience of treatment owing to violence	No	39,501 (74.3)	13,362 (25.7)	39.822 (< .001)
	Yes	363 (62.9)	231 (37.1)	

Data are expressed as weighted mean ± standard error or weighted percentage (%).

^a^*SE* = standard error.

### Predictors of smartphone overdependence

In the complex sample logistic regression model, age, gender, household economic status, anxiety, loneliness, depressive symptoms, and experience of treatment owing to violence were significant predictors of smartphone overdependence. The adjusted odds ratios (ORs) of smartphone overdependence among participants with mild, moderate, and severe anxiety were 2.257 (95% confidence interval [CI] 2.135–2.387), 2.843 (95% CI 2.618–3.087), and 3.326 (95% CI 2.943–3.759), respectively. The adjusted OR of smartphone overdependence in participants who often or always felt lonely was 1.209 (95% CI 1.130–1.295) compared to participants who did not feel lonely or sometimes felt lonely. The adjusted OR of smartphone overdependence among participants experiencing depressive symptoms was 1.187 (95% CI 1.121–1.257) compared to those without such symptoms. Regarding the experience of treatment owing to violence, the OR was 1.254 (95% CI 1.041–1.511) compared to those without experience (Table 3).

**Table 3:** Multiple logistic regression model of factors associated with smartphone overdependence.

Variable (ref^a^)	Categories	OR^b^	95% CI^c^
Age	–	1.062	1.047–1.077
Gender (ref: boys)	Girls	1.344	1.271–1.420
Household economic status (ref: high)	Low	1.166	1.087–1.249
	Medium	1.091	1.040–1.143
Anxiety (ref: minimal anxiety)	Mild	2.257	2.135–2.387
	Moderate	2.843	2.618–3.087
	Severe	3.326	2.943–3.759
Loneliness (ref: none or sometimes)	Often or always	1.209	1.130–1.295
Depression (ref: no)	Yes	1.187	1.121–1.257
Experience of treatment owing to violence (ref: no)	Yes	1.254	1.041–1.511

^a^Reference group, ^b^OR odds ratio, ^c^CI confidence interval.

### Risk subgroups of smartphone overdependence

**Figure 1:**
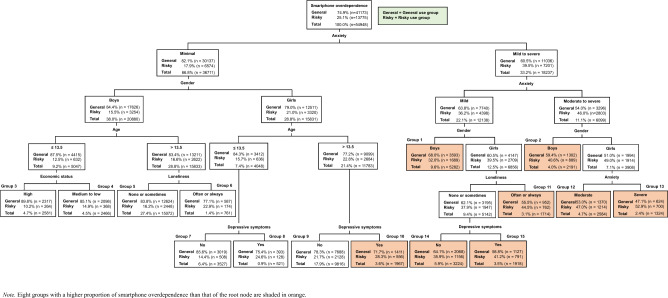
Decision tree analysis results of smartphone overdependence among adolescents.

As a result of the DT analysis, 15 groups were derived, and in eight of these groups (Groups 1, 2, and 10–15), the proportion of smartphone overdependence was higher than that of the root node (shaded in orange in Fig. 1). Specifically, the smartphone overdependence rate of the eight groups ranged from a minimum of 28.3% (Group 10) to a maximum of 52.9% (Group 13), while that of the root node was 25.1%. Six factors including anxiety, gender, loneliness, depressive symptoms, age, and economic status were used to derive the subgroups. Among them, anxiety, gender, loneliness, and depressive symptoms were important in distinguishing the risk subgroups. The group with the highest risk of smartphone overdependence was Group 13, consisting of girls with severe anxiety, and the smartphone overdependence proportion was 52.9%.

## Discussion

This study revealed that anxiety is a significant predictor of smartphone overdependence. The ORs for the risk of smartphone overdependence among adolescents with severe, moderate, and mild anxiety were 3.326, 2.843, and 2.257 times higher than those among adolescents with minimal anxiety, respectively. In addition, in the DT analysis, anxiety was used as the most important factor in distinguishing risk subgroups of smartphone overdependence. We used the GAD-7 for assessing generalized anxiety disorder, which refers to persistent anxiety and uncontrollable worry that persists for more than six months^25^. Previous studies have reported an association between smartphone overdependence and general anxiety^9,10^. Regarding the mechanism of the association between anxiety and smartphone overdependence, individuals experiencing anxiety may turn to their smartphones as a way to escape from stress and avoid confronting anxiety-inducing situations^26,27^. In Korea, the Ministry of Education conducts annual assessments on the emotional and behavioral characteristics of adolescents, collaborating with specialized organizations to support high-risk groups^28^. Based on the findings of this study, anxiety assessment, as well as the prevention and management of smartphone overdependence, must be strengthened in youth mental health management.

Adolescents who were always or often lonely had an approximately 21% higher risk of smartphone overdependence than those who were sometimes lonely or not lonely. The results of the DT analysis showed that girls who experience loneliness have a high risk of falling into smartphone overdependence even if their anxiety is mild. Loneliness refers to the psychological discomfort stemming from the perception that the quantity or quality of one’s actual social relations does not meet one’s needs or desired social relations^29^. These data were collected during the COVID-19 pandemic, when social interactions decreased owing to social distancing measures. In particular, young people were more affected by social distancing because their needs for social relations are high^30^. Importantly, according to statistics from a pre-COVID-19 study performed in Europe, around 12% of European Union citizens reported feeling lonely, while during the pandemic this proportion reached 25%^31^. Adolescents may have felt lonelier because the gap between reduced social relations due to social distancing and their desire for social relations widened during the COVID-19 pandemic. This is supported by the findings of a study during the pandemic showing that young individuals and those reporting lower social support were at a higher risk of loneliness^32^.

The model of pathological Internet use proposes that individuals who are socially isolated and/or lack social support are more likely to waste time on the Internet or spend a great deal of time in chat rooms for no specific purpose. For socially isolated individuals, the Internet can be their only lifeline to the outer world^8^. This can be applied to the phenomenon of smartphone overdependence in the current trends. Individuals who feel lonely owing to social isolation and/or lack of social support may spend extensive periods of time on social media platforms for no specific purpose and might well rely on online relationships forged via smartphones. Consistent with our results, a previous study reported that adolescents who experienced greater social isolation during the COVID-19 pandemic felt lonely, which influenced smartphone overdependence^11^. Another past study reported that people use smartphones to meet the unmet need for social interaction in their daily lives^33^. Furthermore, previous systematic reviews and meta-analyses have demonstrated the effectiveness of psychological interventions, including social skills related to relationship building, personal skills, friendship-related skills, mindfulness, and peer-based social programs, in reducing loneliness. However, they have also presented the need for additional analysis of moderators that might affect effectiveness, such as the intervention length and delivery format (group v. individual and face-to-face v. online)^34^. Therefore, this must be investigated through additional research in the future.

The results of logistic regression analysis showed that the risk of smartphone overdependence in adolescents with depressive symptoms was 1.187 times higher than in adolescents without such symptoms. This finding is consistent with previous studies reporting an association between depression and smartphone overdependence ^9,10,35^. The cognitive–behavioral model of pathological Internet use suggests that depression develops in individuals with problematic Internet use^8^. Additionally, a compensatory Internet use model suggests that individuals use the Internet to alleviate dysphoric moods^36^, while a previous study indicates that individuals with depression are more inclined to engage in smartphone overdependence to alleviate negative moods^37^. Given this evidence, it can be concluded that individuals with depression are overdependent on smartphones as a coping mechanism to alleviate their depressed mood. However, smartphone overdependence alters one’s lifestyle, such as eating unhealthy foods, gaining weight, and developing sleep problems, which can lead to depression^35^. Therefore, providing effective interventions, such as website and mobile application-based interventions, is necessary for adolescents with smartphone overdependence and depression to alleviate these issues^38^. Additionally, to improve service accessibility, policies must be strengthened to enable depression assessment and intervention provision at school health sites.

The experience of treatment owing to violence was associated with smartphone overdependence among adolescents. In previous studies, domestic violence and harsh parenting have been suggested as factors associated with smartphone or Internet addiction^14,39^. Additionally, relationships with peers indirectly affected smartphone overdependence^15^. Thus, when intervening to improve smartphone overdependence, in-depth screening of relationships with parents or friends, experiences of violence, and mental health problems including depression must be conducted. Meanwhile, the experience of treatment owing to violence was included in the analysis owing to the limitations of variables related to the experience of violence among adolescents included in the data; therefore, follow-up studies including additional variables are needed.

Our results indicate that age, gender, and household economic status are associated with smartphone overdependence. Similar to the results of this study, the pattern of problematic smartphone use gradually increased with age in the trajectory analysis results of a study on Chinese adolescents^40^. These findings indicate the need to prevent smartphone overdependence in early adolescence through appropriate interventions. Additionally, consistent with previous findings^4,5^, this study showed that girls had a higher risk of smartphone overdependence than boys. Another past study reported that using smartphones mainly for social media was a predictor of smartphone overdependence among women, whereas playing smartphone games was a predictor among men^41^. Gender differences in smartphone overdependence may be attributed to differences in the main purpose of using smartphones. Further research is needed to examine gender differences, the purpose of using smartphones, and smartphone overdependence.

Adolescents with low household economic status were at a higher risk than those with medium and high household economic status. The smartphone ownership rate of adolescents in South Korea was 98.4% in 2020^42^, indicating that almost all adolescents owned smartphones, regardless of their economic status. Therefore, it is necessary to determine the reasons for the high rate of smartphone overdependence among adolescents with low economic status. Previous research has shown that it is difficult for adolescents with low economic status to choose and enjoy various leisure activities owing to the burden of costs^43^. Future research should examine whether differences in adolescents’ hobbies or activities and parental management according to economic status affect smartphone overdependence.

We used nationally representative data, which allowed the results to be generalized to middle- and high-school student populations across South Korea. The KYRBS previously included variables such as depression, anxiety, and loneliness, but smartphone overdependence was added as a new survey item in 2020. Therefore, studies on smartphone overdependence using these data are scarce. The current work was able to identify the psychological factors of smartphone overdependence using these data. However, this study had several limitations. First, owing to it being a secondary data analysis, the study did not include other psychological variables that may be associated with smartphone overdependence. Specifically, in this study psychological variables were extracted from the KYRBS data based on the cognitive–behavioral model of pathological Internet use^8^; thus variables such as substance use that were not included in the data could not be included in the analysis. Future research must account for this and include various influencing factors of smartphone overdependence. Second, although this survey employed validated and reliable measurements of anxiety and smartphone overdependence, it used simple questions regarding loneliness and depression. However, according to the 2021 regular assessment report published by Statistics Korea, the 2020 KYRBS data remain reliable^20^. Third, the cross-sectional design provided insights into the factors influencing smartphone overdependence but could not establish causal inferences. Fourth, we used self-reported data; therefore, the results could have been affected by social desirability bias. However, disclaimers regarding the protection of anonymity may have encouraged honest responses.

## Conclusion

We found that anxiety, loneliness, depressive symptoms, and treatment experiences owing to violence were predictors of the risk of smartphone overdependence among South Korean students aged 12–18 years. Therefore, early detection and prevention of these issues will be beneficial for preventing smartphone overdependence. Healthcare providers in schools and community mental health centers should make efforts to screen and address adolescents’ anxiety, loneliness, depressive symptoms, and experience of treatment owing to violence. In particular, interventions for smartphone overdependence ought to highlight the assessment, prevention, and treatment of anxiety because of the strong association between anxiety and smartphone overdependence.

## Data Availability

The data that support the findings of this study are openly available from the Korea Disease Control and Prevention Agency at https://www.kdca.go.kr/yhs/, reference number^21^.
